# Primary care is the frontline for help-seeking insomnia patients

**DOI:** 10.1080/13814788.2021.1960308

**Published:** 2021-10-11

**Authors:** Isabel Torrens Darder, Rosmary Argüelles-Vázquez, Patricia Lorente-Montalvo, Maria del Mar Torrens-Darder, Magdalena Esteva

**Affiliations:** a Majorca Primary Care Department, Calviá Primary Health Center, Balearic Islands, Spain; b Health Research Institute of the Balearic Islands (IdISBa), Balearic Islands, Spain; c Majorca Primary Care Department, Muntanya Primary Health Center, Balearic Islands, Spain; d Teaching Unit of Family and Community Medicine, Balearic Islands, Spain; e Preventive Activities and Health Promotion Network (RedIAPP), Balearic Islands, Spain

**Keywords:** Insomnia, primary care, help seek, general practitioner, health care, nursing

## Abstract

**Background:**

Although insomnia is a very common disorder, few people seek medical help.

**Objectives:**

To determine the proportion of people who consult a healthcare professional about insomnia and examine reasons for help seeking.

**Methods:**

Descriptive study of 99 patients diagnosed with insomnia following a telephone survey of 466 adults assigned to a primary healthcare unit in Majorca (Spain). Data were obtained from interviews and subsequent review of electronic medical records.

**Results:**

Thirty-nine patients (39.8%) consulted at least once with one health care professional; 36(92.2%) consulted a general practitioner. Only 12.2% had an insomnia diagnosis registered in their medical record. Insomnia consultation was not associated with any sociodemographic variables analysed, anxiety, depression or comorbidities. Also, there was no association with sleep quality, duration, and sleep efficiency. Patients with clinical insomnia (OR, 2.48; 95% CI, 1.03–5.94), those who were more worried (OR, 2.93; 95% CI 1.08–7.95) or felt that others noticed the impact of insomnia on their quality of life (OR, 2.48; 95% CI, 1.02–19.08) are more likely to seek medical help. Patients taking sleep medication were 21.54 (95% CI, 7.34–63.20) times more likely to have asked for medical assistance.

**Conclusion:**

Insomnia is an under-reported problem for both patients and doctors. When patients decide to consult for insomnia problems, they first go to the GP, and the vast majority take medications for their sleep problem. Those who consult most are people with more severe insomnia and those who are more worried.

Key messages

- Although insomnia is a very common disorder, less than half of patients consult a physician; when they consult, patients contact their general practitioner
- Presence of clinical insomnia is the main trigger for help seeking
- Most of the patients who have consulted are taking insomnia medication

## Introduction

Insomnia is the most common sleep disorder, with prevalence rates ranging from 5 to 48% depending on the definitions and diagnostic criteria used by the different studies [1–6]. Insomnia can have significant daytime consequences, such as irritability, depression, concentration problems and chronic illness, and is associated with increased health services use, drug and alcohol consumption, work absenteeism, and accidents [7–9]. Nevertheless, even though effective cognitive-behavioural and pharmacological treatments exist [10,11], just 27–48% of insomniacs seek help from healthcare professionals [1,12,13].

The most common reason for patients not seeking medical help is the perception of insomnia as a benign and unimportant condition, as they need to deal with on their own [14], and asking for medical help is often their last option [15]. Other common barriers to help-seeking are a lack of awareness of available treatments and a belief that existing treatments are ineffective or unattractive [14]. By contrast, factors found to be associated with help-seeking behaviour are consequences of more severe forms of insomnia such as fewer hours of sleep at night and interference with daytime functioning [12,16], and expectations of a prescription that could relieve patient's insomnia symptoms [15,17].

The primary care setting is a critical venue for identification and early intervention to manage insomnia effectively but patients who do decide to seek medical help for insomnia have greater faith in specialists such as psychiatrists and psychologists than in general practitioners (GPs) or nurses [18]. A common perception is that GPs’ knowledge about insomnia is limited to offering advice on sleep hygiene and prescribing drugs while specialists are seen as more knowledgeable and have a better understanding of the nature of the problem and treatment approaches [19].

This study aimed to investigate how many patients with insomnia sought help from a professional, to know which professional they initially contacted and compare sociodemographic characteristics, comorbidities and sleep quality between those who contact a primary care health professional and those who did not.

## Methods

### Study design

The present study forms part of a broader descriptive cross-sectional survey, investigating the prevalence of insomnia and patient characteristics in the primary care health centre of Calviá (Majorca, Spain) with a population of 23,200 inhabitants registered.

The study was carried out during 2010–2011 and insomnia prevalence results have already been published [6]. The study was developed through two phases. Phase 1 was a telephone insomnia screening survey to detect the population with persistent insomnia. Participants were a random sample generated with Epidat v3.1 of 1563 individuals, aged between 18 and 80 years, registered in the health centre for more than two years. Exclusion criteria were pregnancy, restless legs syndrome, sleep apnoea, narcolepsy, attention-deficit/hyperactivity disorder, palliative care, severe cognitive deficit, psychosis and major depression. Diagnosis of insomnia was based on the Insomnia Severity Index (ISI) score of ≥8.

Participants with detected insomnia were invited to participate in a second phase based on an interview in the health centre. These individuals formed the sample for the present study. This study received the approval of the Majorca Primary Care Ethical Research Committee PI11/15.

### Measures

The survey participants with insomnia detected get an appointment at the health centre with a trained nurse or GP. During the interview, all participants were given oral and written information about the study and provided signed consent. The following data were collected: (a) sociodemographic data, (b) the presence of comorbidities, using the list of the Spanish Health Survey [20], (c) any visit to a GP or other healthcare professional about insomnia, (d) use of sleep medication, (e) anxiety and depression measured with the 14-item Spanish version of the Hospital Anxiety and Depression Scale (HADS) [21], (f) insomnia duration, (g) insomnia severity; measured with the Spanish version of the Insomnia severity Index (ISI) [22], which elicits 0–4 severity ratings (‘none’ to ‘very’/’very much’) for recent problems with sleep. A composite score is obtained by adding ratings given to the different items (total possible score, 0–28). Higher scores indicate more severe insomnia [22,23]. According to the ISI scores, patients were classified as sub threshold 8–14 and clinical insomnia >14, and h) Quality of sleep was measured using the Spanish version of the Pittsburgh Sleep Quality Index (PSQI) [24,25], which contains 19 items that assess seven self-reported clinically relevant components of sleep quality in the preceding month: subjective sleep quality, sleep latency, sleep duration, sleep efficiency, sleep disturbances, use of sleep medication, and daytime dysfunction. The first four items are answered by free entry, while the rest are rated with a score ranging from 0 to 3, where 0 indicates no problems and 3 indicates a severe problem. A cut-off of 5 discriminates between good and bad sleepers.

After the interviews, the patients’ electronic medical records were reviewed to collect the following information registered during five years before patient inclusion in the study: (a) any visit with a GP or practice nurse about insomnia; (b) any visit with a mental health specialist (psychologist or psychiatrist) about insomnia; (c) a diagnosis of insomnia registered in the patient’s medical record; (d) prescription of pharmacological treatment for insomnia.

### Statistical analysis

A descriptive analysis was performed using frequencies and percentages to describe categorical variables. The participants’ rate who had sought medical help for insomnia was calculated. Associations between sociodemographic variables, number of comorbidities (grouped as 0–2 or ≥3), sleep quality, and insomnia consultations were estimated using the χ2 test and calculation of odds ratios with 95% CIs. Statistical significance was considered at a *p* value < 0.05. Analyses were performed with SPSS v. 23.

## Results

**Figure 1.**
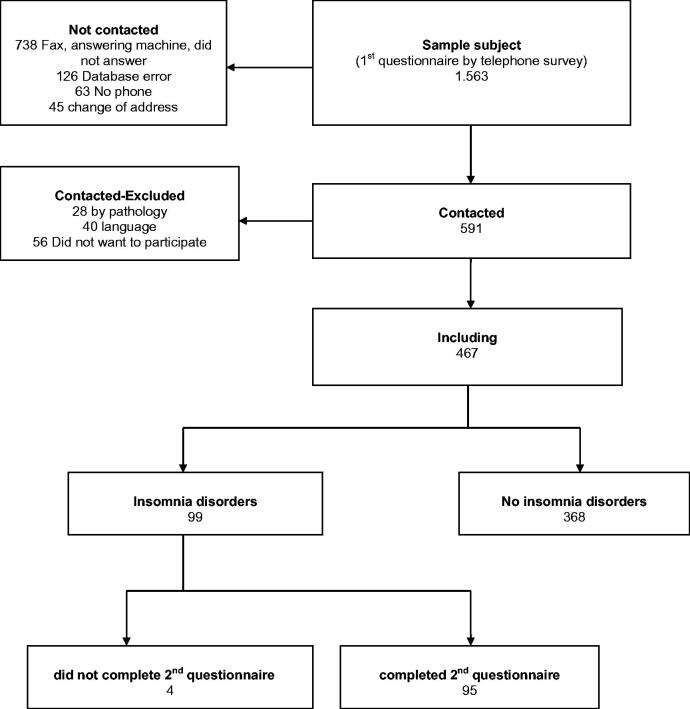
Flowchart of inclusion of subjects in the study.

The flowchart of inclusion of the study population is shown in Figure 1. Of the 467 who answered the first phase, 99 patients were diagnosed with insomnia (ISI score ≥8) and 31 (31.3%) of them were classified as having clinical insomnia (ISI >14). We were unable to access the medical record of one patient who had moved out of Majorca. Patients’ characteristics with insomnia have already been published [6]. These were predominantly women, patients with a stable partner, primary school education, those who had a job, and those younger than 65 years (Table 1).

**Table 1.** Sociodemographic characteristics of sample with insomnia (*n* = 99).

	No. (%)
Sex	
Male	30 (30.3)
Female	69 (69.7)
Civil status	
Single	22 (22.2)
Stable partner	54 (54.5)
Separated/divorced/widowed	18 (18.2)
Do not know/no answer	5 (5.1)
Employment status	
Employed	44 (44.4)
Unemployed	21 (21.2)
Retired	25 (25.3)
Student	0 (0.0)
Homemaker	3 (3.0)
Do not know/no answer	6 (6.1)
Level of education	
Illiterate/incomplete primary studies	32 (32.3)
Primary	23 (23.2)
Secondary	24 (24.2)
University	13 (13.1)
Do not know/no answer	7 (7.1)
Age, y	
<50	45 (45.5)
50–65	40 (40.4)
>65	14 (14.1)

### Help-seeking behaviour

Based on the review of the medical records, 39 patients (39.8%, 95% CI 29.5–48.9%) had discussed their insomnia with a healthcare professional. The vast majority (*n* = 36, 92.2%) had consulted a GP; 8 (20.6%) had consulted a psychologist and 3 (7.8%) a psychiatrist. None had consulted a practice nurse. Of those who visited a health professional, four patients (10.3%) had discussed their insomnia with at least two healthcare professionals while 3 (8%) had consulted three (GP, psychologist, and psychiatrist). Forty-five patients (45.9%) had been prescribed sleep medication. Just 12 patients (12.2%) had a diagnosis of insomnia registered in their medical record.

### Characteristics of people looking for help

More women than men sought medical help for their insomnia, although the difference was not significant (Table 2). No significant differences were observed either for age, civil status, or level of education. Help-seeking behaviours were more common in patients with anxiety (but not statistically significant) than in those with depression or more medical conditions.

**Table 2.** Association between help seeking for insomnia and sociodemographic factors, number of comorbidities, and the presence of anxiety and depression.

	**Consultation** **No. (%)**	**Non consultation** **No. (%)**	OR (95% CI)	P
Sex				
Male	8 (20.5)	22 (37.3)	1	
Female	31 (79.5)	37 (62.7)	2.30 (0.90–5.85)	0.07
Age, (years)				
< 40	6 (15.4)	16 (27.1)	1	
40–49	11 (28.2)	12 (20.3)	2.44 (0.70–8.48)	0.15
50–64	14 (35.9)	22 (37.3)	1.69 (0.53–5.37)	0.36
≥ 65	8 (20.5)	9 (15.3)	2.37 (0.62–9.02)	0.20
Civil status				
Single	8 (21.6)	14 (25.0)	1	
Stable partner	19 (51.4)	35 (62.5)	0.95 (0.33–2.66)	0.92
Divorced/widowed	10(16.2)	7 (12.5)	2.50 (0.68–9.16)	0.16
Level of education				
Illiterate/incomplete primary studies	12 (34.3)	19 (33.9)	1	
Primary	8 (22.9)	15 (26.8)	0.84 (0.27–2.59)	0.76
Secondary	10 (28.6)	14 (25.0)	1.13 (0.38–3.35)	0.82
University	5 (14.3)	8 (14.3)	0.99 (0.26–3.74)	0.98
Employment status				
Working	19 (52.8)	25 (44.6)	1	
Unemployed	8 (22.2)	13 (23.2)	0.81 (0.27–2.34)	0.15
Retired/student/not working	9(32.3)	18 (32.1)	0.65 (0.24–1.78)	0.41
Comorbid conditions, No.				
0–2	18 (48.6)	32 (34.8)	1	
≥3	19 (51.4)	23 (41.8)	1.46 (0.63–3.39)	0.39
Depression				
No	26 (70.3)	46 (83.6)	1	
Yes	11 (29.7)	9 (16.4)	2.16 (0.79–5.89)	0.12
Anxiety				
No	18 (58.6)	38 (69.1)	1	
Yes	19 (51.4)	17 (30.9)	2.35 (0.99–5.58)	0.05

Results of ISI and PSQI are summarised in Table 3 according to whether patients consulted or not about their insomnia. Patients with clinical insomnia (ISI ≥14), those who were worried about their insomnia, and those who felt that the effects of their insomnia on quality of life were noticeable to others were significantly more likely to consult a healthcare professional.

**Table 3.** Association between help seeking for insomnia and insomnia severity and sleep quality.

	Consultation No. (%)	Non consultation No. (%)	OR (95% CI)	P
Insomnia duration (months)				
< 12	5 (13.5)	9 (16.4)	1	
≥12	32 (86.5)	46 (83.6)	1.25 (0.38–4.08)	0.70
Insomnia severity Index				
Subthreshold	22 (56.4)	45 (76.3)	1	
Clinical	17 (43.6)	14 (23.7)	2.48 (1.03–5.94)	0.03
Worried				
Not at all/a little	11 (42.4)	25 (23.1)	1	
Somewhat	9 (27.1)	16 (28.2)	1.19 (0.64–5.63)	0.24
Very/very much	19 (30.5)	18 (30.5)	2.93 (1.08–7.95)	0.03
Interference with daily functioning				
Not at all/a little	6 (15.4)	16 (27.1)	1	
Somewhat	14 (35.9)	26 (44.1)	1.43 (0.45–4.49)	0.53
Much/very much	19 (48.7)	17 (28.8)	2.98 (0.94–9.35)	0.61
Noticeable to others				
Not at all/a little	19 (48,3)	36 (61.0)	1	
Somewhat	13 (33,3)	20 (33.9)	1.23 (0.50–3.00)	0.64
Much/very much	7 (17.9)	3 (5.1)	4.42 (1.02–19.08)	0.04
Satisfaction				
Very satisfied/satisfied	5 (8,5)	4 (10.3)	1	
Moderately satisfied	21 (35,6)	6 (15.4)	0.35 (0.07–1.72)	0.20
Dissatisfied/very dissatisfied	33 (55.9)	29 (74.4)	1.09 (0.26–4.48)	0.09
Sleep efficiency (%)				
<85	9 (24.3)	15 (27.3)	1	
75–85	5 (13.5)	10 (18.2)	0.83 (0.21–3.23)	0.79
65–75	7 (18.9)	8 (14.5)	1.45 (0.39–5.39)	0.57
<65	16 (43.2)	22 (40.0)	1.21 (0.42–3.45)	0.71
Sleep duration (hours)*				
>7	0 (0)	0 (0)	1	
6–7	17 (48.5)	26 (49.0)	0.77 (0.17–3.41)	0.73
5–6*	9 (25.7)	16 (30.2)	0.70 (0.15–3.30)	0.65
<5	9 (25.7)	11 (20.8)	1.02 (0.21–4.97)	0.98
Sleep quality				
Good/very good	7 (18.9)	42 (76.4)	1	
Poor/very poor	30 (81.1)	13 (23.6)	1.32 (0.47–3.72)	0.59

Patients who had been prescribed sleep medication formed the largest proportion of patients who sought medical help for their insomnia (84.6%) and they were over 20 times more likely than patients not on prescribed medication (OR, 21.54; 95% CI, 7.34–63.20). A Kappa value of 0.49, indicating moderate agreement, was observed between self-reported medication use and sleep prescription records in clinical notes.

## Discussion

### Main findings

Our results show that just four of every 10 people with insomnia seek help for their problem from healthcare professionals. Help-seeking behaviour in our population was significantly associated with clinical insomnia (ISI >14). Patients were also more likely to seek professional help if they were worried about their insomnia or if they felt that its impact on their quality of life was noticeable to others. People in these situations have probably come to recognise that their insomnia is a relevant health problem. Most of the patients who consulted a healthcare professional about their insomnia were taking sleep medication. This could be because doctors frequently are more prone to prescribe pharmacological drugs to treat insomnia or because patients’ main reason for visiting their doctor is to request medication that may relieve their insomnia symptoms. Most help seekers in our study had consulted a GP, but this could be due to the nature of the Spanish public healthcare system, where patients can only gain access to other specialists through their GP. It is therefore likely that GPs only referred more complicated cases to mental health specialists.

### Strengths and limitations

This was a population-based study where information on insomnia prevalence was obtained by telephone interviews with a random sample of the population assigned to a healthcare centre [6]. We think it would provide a more reliable estimate of the prevalence of insomnia in the general population than studies that calculate prevalence based on members of the population who consulted with a primary healthcare professional. This approach has also been problematic since we had many difficulties in contacting patients, resulting in a low response rate in the prevalence survey and in consequence a low sample size of population with insomnia. We estimated percentages of consultation through medical records that could be affected by information bias, as sleep problems are often not the main reason for consultation and as such might be underreported. Our results show that most GPs have recorded insomnia symptoms declared during a medical consultation as text notes but not as a diagnosis. This fact could result in a lesser follow-up of the problem and less probability of the sleeping disorder being solved.

### Comparison with existing literature

Overall, 39.8% of the patients with insomnia sought medical help for their problem; this figure is similar to others reported in the literature (40–48%) [12,26,27], which also showed more than half of patients with insomnia did not receive any help from a professional. Even some of those patients with clinical insomnia and interference in daily functioning did not reach clinical attention. All studies analysed, reported that patients with insomnia were much more likely to consult a GP rather than another specialist, although the rate in our study population, 92.2%, is somewhat higher than rates reported by Morin et al., (82.7%) [27], Stinson et al., (72.9%) [14] and Cheung et al., (65%) [19]. This could be because in Spain, access to psychiatrists and psychologists is through GP referral. We observed that insomnia in our population was under-registered and under-diagnosed by GPs as observed by others studies probably because it was considered as a trivial or temporary problem or because it has been reported to the GP together with other conditions. It was not given priority in the patient agenda [18,28]. As the review of Araujo et al., highlights [29], there is a mismatch between patients’ and health care professionals’ points of view on the experience of insomnia, as well as the complexity and extent of the phenomenon. Health professionals tend to give less attention to the subjective experience of insomnia resulting in under-diagnosis of the insomnia complaints [28].

Women were more likely to seek help from a healthcare professional than men but the difference was not significant, as also seen by Liu et al., and Morin et al., [26,27]. Discrepancies were also noted for sociodemographic variables. While significant associations have been reported between insomnia consultations and older age [30], some college education [12] and unemployment [27], these factors were not significant in our participants.

Our results for anxiety and depression also differ from those reported in the literature. Patients with anxiety were more likely to consult a professional about their insomnia (although not statistically significant), but this was not the case for those with depression, contrasting with findings by other authors who have reported a significant association between consultations and depression [30] and psychiatric disorders in general [26,31]. Also in contrast to other studies [12,26], patients with more medical conditions were not significantly more likely to seek help for their insomnia.

One of the most consistent reasons for seeking help is the severity of insomnia and how it affects daily life activities. In agreement with Liu et al., [26], we found that patients with clinical insomnia (ISI >14) and sleep medication users were more likely to have consulted about their problem. Also, patients more worried about insomnia and those with daily life impact noticeable to others were more prone to seek medical help. Findings of Cheung et al., in specialist care [19], show that daytime symptoms arising from insomnia serve as important illness cues for patients to seek medical help. Others found that non-restorative sleep and difficulties initiating sleep were the factors most strongly associated with a self-reported need for the treatment of sleeping difficulties [31].

### Implications for clinical practice

Despite the considerable impact of insomnia on the quality of daily life of those suffering, it is still an under-reported health problem. Those who seek medical help mainly contact their primary care physician. Continuity of care in primary care is a unique opportunity to recognise insomnia during clinical interviews. Being more active and asking more assertive sleep-related questions in patients with sleep complaints during a consultation for other conditions could be a helpful approach in detecting insomnia. Since many persons who have insomnia do not consult, it would be advisable to design strategies aimed at community care, either to detect insomnia or for its management. Other studies are needed in this field, especially with a qualitative approach to explore both professionals and patients’ perspectives on insomnia as a health disorder and beliefs around its management.

## Conclusion

Insomnia is an under-reported problem for both patients and doctors. When patients decide to consult for insomnia problems, they first go to the GP, and the vast majority of them take medications for their sleep problem. Half of the patients do not request medical help and consequently, a vital percentage that have clinical insomnia or who are worried about the disorder miss the opportunity to receive a treatment that could benefit them.
